# Non-invasive intraductal oncocytic papillary neoplasm forming a protruding lesion toward the duodenum from the accessory papilla: a case report

**DOI:** 10.1186/s40792-024-01841-w

**Published:** 2024-02-15

**Authors:** Shinnosuke Kawahara, Naoto Yamamoto, Kota Washimi, Rei Kanemoto, Daishi Takahashi, Yuto Kamioka, Itaru Hashimoto, Mariko Kamiya, Aya Kato, Yukio Maezawa, Keisuke Kazama, Masaaki Murakawa, Sho Sawazaki, Toru Aoyama, Hiroshi Tamagawa, Takashi Oshima, Norio Yukawa, Yasushi Rino, Tomoyuki Yokose, Aya Saito, Soichiro Morinaga

**Affiliations:** 1 Department of Gastrointestinal Surgery, Kanagawa Cancer Center, 2-3-2 Nakao, Asahi-ku, Yokohama, 241-8515 Japan; 2 Department of Pathology, Kanagawa Cancer Center, 2-3-2 Nakao, Asahi-ku, Yokohama, 241-8515 Japan; 3 Department of Surgery, Yokohama City University, 3-9 Fukuura, Kanazawa-ku, Yokohama, 236-0004 Japan

**Keywords:** Intraductal oncocytic papillary neoplasm, Pancreas, Accessory papilla, Non-invasive

## Abstract

**Background:**

Intraductal oncocytic papillary neoplasm (IOPN), previously classified as a subtype of intraductal papillary mucinous neoplasm (IPMN), has been described as an independent disease by the WHO since 2019. IOPN is a rare tumor, with few reported cases. Herein, we report a case of resected non-invasive IOPN that formed a lesion protruding toward the duodenum from the accessory papilla.

**Case presentation:**

An 80-year-old woman was referred to our hospital because of a giant mass in the pancreatic head detected on abdominal contrast-enhanced computed tomography (CT) performed for a close examination of a mass in the right breast. CT revealed a 90-mm-sized tumor with a mixture of solid and cystic components, with contrast enhancement in the pancreatic head, and a dilated main pancreatic duct. Esophagogastroduodenoscopy revealed a semi-circumferential papillary tumor protruding toward the duodenal lumen, which did not protrude from the papilla of Vater. Transpapillary biopsy led to a preoperative diagnosis of IPMN with an associated invasive carcinoma. As there were no distant metastasis, open subtotal stomach-preserving pancreaticoduodenectomy was performed. Analysis of the surgical specimen and histopathological examination revealed that the tumor was an IOPN that protruded toward the duodenal mucosa from the accessory papilla while replacing the duodenal mucosa with no obvious stromal invasion.

**Conclusion:**

IOPN is a rare and poorly recognized tumor with few reported cases. There have been no reports describing IOPN forming a protruding lesion toward the duodenum from the accessory papilla. Therefore, further accumulation of cases such as this one is important to advance the study of IOPN.

## Background

Intraductal oncocytic papillary neoplasm (IOPN), rare neoplasms arising in the pancreas and biliary tree, was first reported by Adsay et al. in 1996 [1]. IOPN was previously classified as an oncocytic subtype intraductal papillary mucinous neoplasm (IPMN). However, IOPN has been described as a disease concept independent of IPMN by the WHO since 2019 (WHO Classification of Tumors, 5th edition) [2, 3].

IOPN is a rare tumor, accounting for only 4.5–8% of IPMN resection cases [4, 5], and reports of IOPN remain rare. Herein, we report a rare case of resected non-invasive IOPN that formed a lesion protruding toward the duodenum from the accessory papilla.

## Case report

**Fig. 1:**
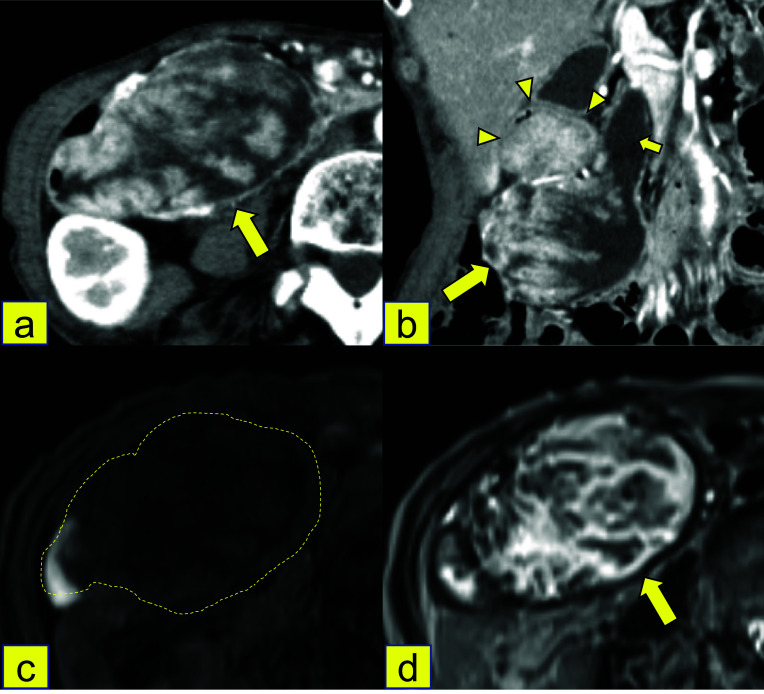
Imaging findings of the patient. **a** Abdominal contrast-enhanced CT in the axial section showed a 90-mm-sized tumor with a mixture of solid and cystic components with contrast enhancement in the pancreatic head (arrow). **b** CT imaging of the coronal section showed the tumor (large arrow) grew within the main pancreatic duct (small arrow), not within branches, compressing and narrowing the duodenum (arrowhead). **c** T1-weighted MRI showed a giant cystic lesion with a clear border and smooth margin in the pancreatic head with low intensity (dotted line). **d** T2-weighted MRI showed a giant cystic lesion with high intensity (arrow)

**Fig. 2:**
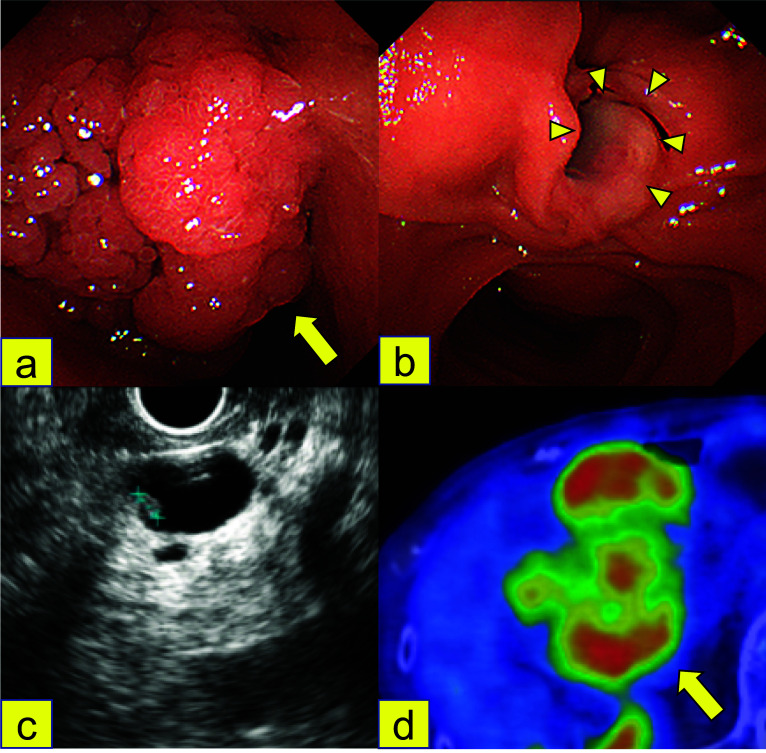
Examination and imaging findings. **a** EGD showed a semi-circumferential papillary tumor protruding toward the duodenal lumen (arrow). **b** The mucosa of the Vater papilla was intact (arrowhead). **c** EUS revealed a flat isoechoic mural nodule 4 mm in diameter in the dilated main pancreatic duct of the pancreatic head. **d** FDG-PET-CT showed an accumulation of SUV max 6.75 in the pancreatic head tumor (arrow)

An 80-year-old woman was referred to our hospital following detection of a giant mass in the pancreatic head on abdominal contrast-enhanced computed tomography (CT) performed for a close examination of a mass in the right breast. She had no gastrointestinal obstruction, no weight loss, no history of diabetes or ovarian cancer, and no family history of cancer. Blood test results showed mild anemia (red blood cells: 368 × 10^4^/mm^3^, hemoglobin: 11.8 g/dl) and hypoalbuminemia (albumin: 3.5 g/dl). Hepatobiliary enzyme and tumor marker levels (carcinoembryonic antigen (CEA): 3.8 ng/ml, carbohydrate antigen 19-9 (CA19-9) 12 U/ml, DUPAN-2 ≤ 25 U/ml, Span-1 19 U/ml) were normal. CT revealed a 90-mm-sized tumor in the pancreatic head comprising a mixture of solid and cystic components, with contrast enhancement (Fig. 1a). The tumor had grown within the main pancreatic duct, not within branches, compressing and narrowing the duodenum (Fig. 1b). Magnetic resonance imaging (MRI) revealed a giant cystic lesion with a clear border and smooth margin in the pancreatic head, contiguous with the dilated main pancreatic duct (MPD). On MRI, the tumor showed as low intensity in the interior on T1-weighted image (T1WI), high intensity in the interior on T2WI, and low intensity at the periphery on both images (Fig. 1c, d). As both CT and MRI suggested that the lesion was located in the pancreatic head invading the duodenum, esophagogastroduodenoscopy (EGD) and endoscopic ultrasonography (EUS) were performed. EGD revealed a semi-circumferential papillary tumor protruding into the lumen from the first portion of the duodenum to the anterior wall of the second portion (Fig. 2a). The tumor was located twofold proximal to the papilla of Vater, and the mucosa of the papilla of Vater was intact (Fig. 2b). EUS revealed a flat isoechoic mural nodule of 4 mm in the dilated MPD in the pancreatic head (Fig. 2c), which was not found in the pancreatic body or tail. Tumor biopsy performed at the time of EGD revealed atypical cylindrical cells with enlarged nuclei in a tubular and papillary arrangement on hematoxylin–eosin (HE) staining, and the pathological diagnosis was adenocarcinoma. Fluorodeoxyglucose-positron emission tomography (FDG-PET-CT) revealed an accumulation of SUV max 6.75 in the pancreatic head tumor, but not in other organs (Fig. 2d).

**Fig. 3:**
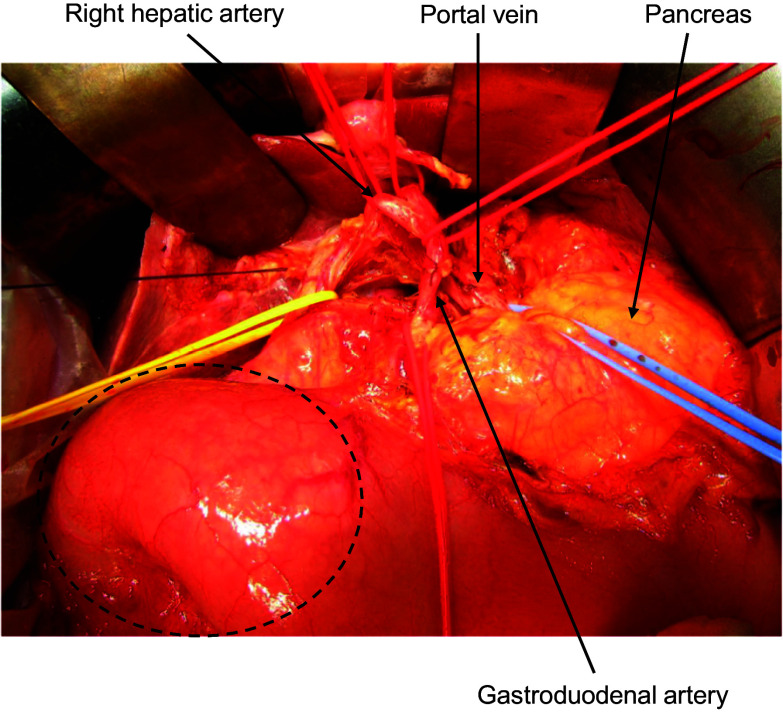
Intraoperative findings. During laparotomy, the tumor was found to be located in the pancreatic head without serosal or vascular invasion (dotted line)

Based on these findings, the patient was diagnosed with main duct-type or mixed-type IPMN with an associated invasive carcinoma. Because of the absence of distant metastasis, although the mass in the right breast was diagnosed as a ductal carcinoma in situ by biopsy, IPMN with an associated invasive carcinoma was considered a prognostic factor by the institutional tumor board, and we therefore performed a curative-intent pancreatic resection. Considering the patient’s age and wishes, surgery was performed without neoadjuvant chemotherapy. Open subtotal stomach-preserving pancreaticoduodenectomy (SSPPD) and regional lymphadenectomy were performed after confirming negative intraoperative peritoneal lavage cytology. During laparotomy, the tumor was identified in the pancreatic head, with no obvious serosal or vascular invasion (Fig. 3). The duration of surgery was 314 min, and intraoperative blood loss was 55 ml. The patient was discharged on postoperative day 13 without complications.

**Fig. 4:**
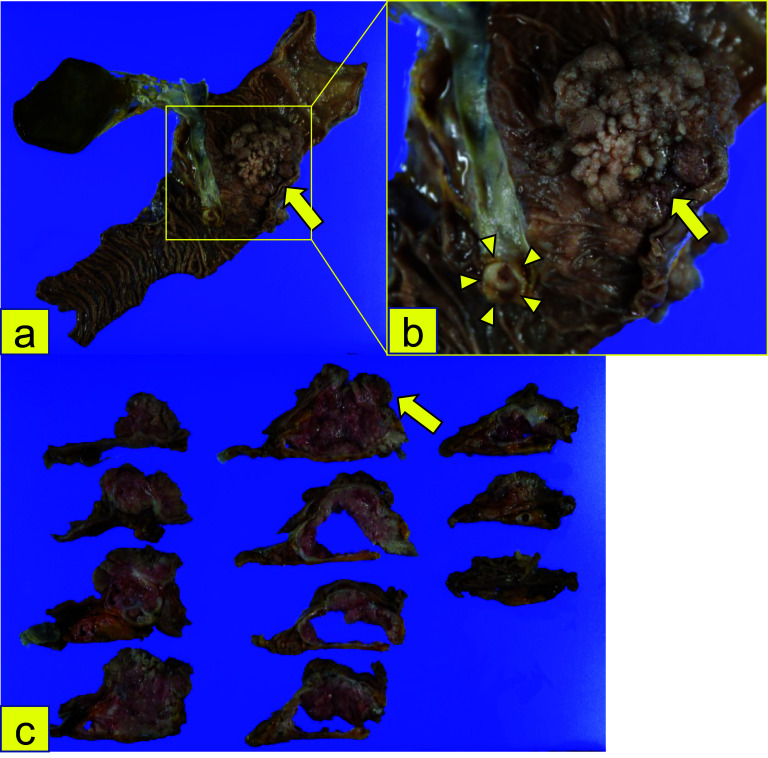
Macroscopic findings of the resected specimen. **a–c** The resected specimen showed a papillary tumor 75 mm in diameter, protruding toward the duodenum from the pancreatic head (arrow). The mucosa of the Vater papilla was intact (arrowhead)

**Fig. 5:**
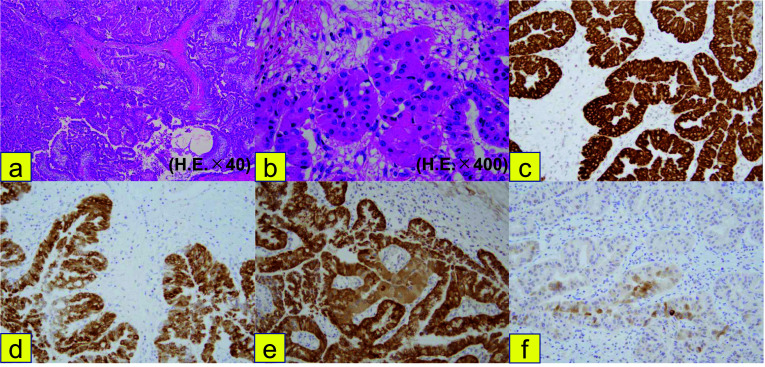
Histological findings of the resected specimen. **a**, **b** Cells with eosinophilic cytoplasm, deeply stained nuclei, and mucus increased papillary were identified on HE staining. **c** MUC5AC, **d** MUC6, and **e** HepPar-1 were positive on immunohistochemical staining. **f** MUC2 was only partially positive

**Fig.6:**
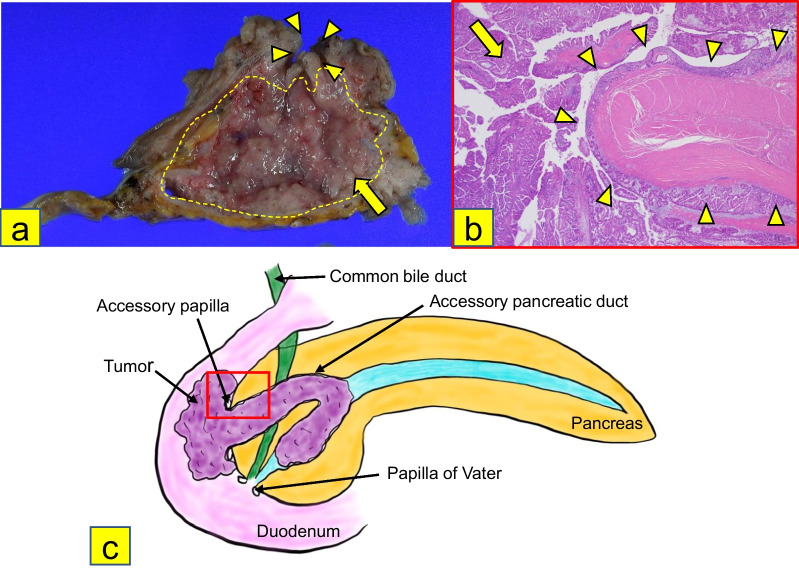
Histological findings around the accessory papilla and the diagram of the resected specimen. **a** The accessory pancreatic duct (dotted line) was filled with the papillary lesions (arrow), and the lesions were protruding toward the duodenal mucosa from the accessory papilla (arrowhead). **b**, **c** The papillary lesions (arrow) were found in the epithelium of both the main accessory pancreatic ducts, and protruded toward the duodenal mucosa from the accessory papilla (arrowhead), while extending to replace the duodenal mucosa, with no obvious stromal invasion

The surgical specimen revealed a papillary tumor with a diameter of 75 mm, protruding toward the duodenum from the pancreatic head. The tumor contained cysts filled with mucus. The mucosa of the papilla of Vater was intact (Fig. 4a–c). Surgical margins were negative (R0). Histopathological examination revealed cells with an eosinophilic cytoplasm, deeply stained nuclei, and mucus with increased papillae on HE staining (Fig. 5a, b). Immunohistochemical staining revealed that the tumor cells were positive for MUC5AC, MUC6, and HepPar-1; and partially positive for CDX-2 and MUC2 (Fig. 5c–f). In addition, papillary lesions were identified in the epithelium of both the main and accessory pancreatic ducts and protruded toward the duodenal mucosa from the accessory papilla, while extending to replace the duodenal mucosa with no obvious stromal invasion on HE staining (Fig. 6a–c). Based on these findings, the patient was pathologically diagnosed with High-grade IOPN. The final pathological stage was TisN0M0, Stage0 (UICC for International Cancer Control, 8th edition), and the patient did not receive adjuvant chemotherapy.

Two months after pancreatectomy, partial mastectomy with sentinel lymph node biopsy was performed for right breast cancer, and the patient was diagnosed with ductal carcinoma in situ. The patient has remained alive 5 months after pancreatectomy, with no recurrence or metastasis.

## Discussion

IOPN, previously classified as an oncocytic-type IPMN, was first reported in 11 cases by Adsay et al. in 1996 [1]. IOPN is a cystic tumor with traffic to the dilated pancreatic ducts due to mucus production and with contrast-enhanced mural nodules, and histologically, unlike IPMN, is characterized by mitochondria-induced eosinophilic granulation of the cytoplasm. Recent investigations of IOPN have reported that KRAS, GNAS, and RNF43 mutations, which are frequently observed in IPMN, are rare, while PRKACA and PRKACB fusion genes and gene mutations in ARHGAP26, ASXL1, EPHA8, and ERBB4, which are not observed in IPMN, have been detected [6–11]. Therefore, IOPN was described first as an independent disease the 2019 WHO guidelines [2, 3]. Accordingly, the Japanese General Rules for the Clinical and Pathological Study of Pancreatic Cancer, 7th Edition, and its reprints were revised in 2020.

IOPN is a rare tumor, accounting for only 4.5–8% of IPMN resection cases [4, 5]. A previous study outlining the features of IOPN revealed the main chief complaints with abdominal pain and discomfort, diabetes as comorbidity, no sex differences, the average age at diagnosis of 61 years (range 36–84 years), the tumors tended to occur in the pancreatic head (64%), the average tumor size of 55 mm (range 10–150 mm), and normal tumor markers levels such as CA19-9, CEA, and DUPAN-2 [1, 11]. Regarding preoperative imaging examinations, CT and MRI of IOPN often reveal a large lesion with polycystic and prominent solid components in the pancreatic head, a dilated pancreatic duct, and contrast-enhanced mural nodules. In addition, similar to pancreatic cancer, FDG-PET/CT may be used to diagnose IOPN and evaluate malignancy and distant metastasis. Noji et al. previously observed a significantly high accumulation of mural nodules on FDG-PET-CT and assumed that this reflected high metabolic activity due to the abundant mitochondria in the IOPN cytoplasm and the high density of tumor cells [12]. In contrast, IOPN was previously classified as an IPMN subtype, and the imaging characteristics of CT, MRI, and PET-CT are similar to those of IPMNs; therefore, it is extremely difficult to diagnose IOPN preoperatively based on radiological imaging examinations [11, 13, 14]. Therefore, preoperative tumor biopsies and pancreatic juice cytology are often performed. If a tumor biopsy or pancreatic juice cytology shows eosinophilic tumor cells, IOPN may be diagnosed preoperatively.

This case has two unique features. First, the giant papillary lesion protruded from the accessory papilla toward the duodenal lumen. Although it was difficult to diagnose whether the tumor originated from the main or accessory pancreatic ducts, we hypothesized that the tumor had traversed along the main and accessory pancreatic ducts, replacing the duodenal mucosa, and extending continuously into the duodenal mucosa, ultimately resulting in extrusion into the duodenal lumen from the accessory papilla. There have been no reports describing IOPN forming a lesion protruding toward the duodenum from the accessory papilla, but some reports of IPMN, a similar neoplasm, with these features have been published [15]. In our case, the giant lesion was exposed in the duodenal lumen, which allowed for a preoperative endoscopic transpapillary biopsy. Among biopsy and cytology techniques, EUS-guided fine-needle aspiration has attracted attention in Europe and the United States, and is useful for the differential diagnosis of cystic tumors through the measurement of fluid content, cytology, amylase, CEA, and CA19-9 [16–18]. Whereas, in Japan, EUS-FNA should not be performed for IPMN as biopsy of the cystic portion increases the risk of tumor cell dissemination [19], and biopsy is often performed from the main pancreatic duct under endoscopic retrograde cholangiopancreatography. However, pathological diagnosis is difficult when the amount of tissue obtained from the biopsy is small; most cases are diagnosed with IPMN, pancreatic ductal adenocarcinoma, or neuroendocrine tumor, and more than 90% are diagnosed with IOPN postoperatively [11]. In our case, although we initially diagnosed the patient as IPMN with an associated invasive carcinoma because of the small amount of tissue, in cases where the lesions protrude toward the duodenal lumen, transpapillary biopsy can be used to diagnose IOPN preoperatively without the risk of dissemination.

Second, although the lesion was large, no obvious stromal invasion was observed. More than 90% of IOPN cases are malignant, comprising invasive and high-grade, i.e., non-invasive carcinoma, while a systematic review by Wang et al. reported that approximately half were classified as non-invasive [11]. Moreover, because even the invasive parts are usually inconspicuous and often confined to the pancreas [20], and lymph node metastasis and tumor death are less common than in other groups of precancerous lesions [4, 14, 21], the prognosis of IOPN is considered superior to that of IPMN. However, the rate of lymph node metastasis in IOPN is at approximately 10%, pancreatic resection with regional lymphadenectomy is recommended in all cases [11]. In addition, there have been some reports of recurrence of liver metastasis even in non-invasive cases [1, 13, 22, 23] and careful follow-up is necessary to monitor postoperative recurrence.

In conclusion, we encountered a case of non-invasive IOPN that formed a giant lesion protruding toward the duodenum from the accessory papilla. Thus far, there have been few published reports of IOPN, and the concept of IOPN is not widely known. Previous studies have focused on the histopathological features of IOPN; whereas, as case series have accumulated, the focus has shifted to the prognosis of IOPN and how it differs from other subtypes of IPMN. Therefore, further accumulation of cases is important to advance IOPN research.

## Data Availability

The authors declare that all the data in this article are available from the corresponding author upon request.

## Abbreviations

IOPN: Intraductal oncocytic papillary neoplasm
IPMN: Intraductal papillary mucinous neoplasm
CT: Computed tomography
CEA: Carcinoembryonic antigen
CA19-9: Carbohydrate antigen 19-9
MRI: Magnetic resonance imaging
MPD: Main pancreatic duct
T1WI: T1-weighted image
EGD: Esophagogastroduodenoscopy
EUS: Endoscopic ultrasound
HE: Hematoxylin–eosin
FDG-PET: Fluorodeoxyglucose-positron emission tomography
SSPPD: Subtotal stomach-preserving pancreaticoduodenectomy
JGR: The Japanese General Rules for the Clinical and Pathological Study of Pancreatic Cancer
R0: Absent of residual tumor
